# Supplementary material for: Dietary variability and micronutrient status of individuals with Yaws infection in Ghana: A case-control study
Source: PLoS One. 2025 Oct 17;20(10):e0334628. doi: 10.1371/journal.pone.0334628 (PMC12533875; doi:10.1371/journal.pone.0334628)
Supplement: S1 Table — (DOCX) [file pone.0334628.s001.docx]

**S1 Table Distribution of intake diversity by sex among participants**

| **Food Group** |  |  | **Food group intake** |  |
| --- | --- | --- | --- | --- |
|  |  | All,  N=128 (%) | Cases  N=64 (%) | Controls  N=64 (%) |
| Grains, roots, and tubers |  | **128 (100)** | **64 (100)** | **64 (100)** |
|  | Male | 95 (100) | 47 (100) | 48 (100) |
|  | Female | 33 (100) | 17 (100) | 16 (100) |
| Green leafy vegetables |  | **45 (35)** | **22 (34)** | **23 (36)** |
|  | Male | 33 (35) | 15 (32) | 18 (38) |
|  | Female | 12 (36) | 7 (51) | 5 (31) |
| Flesh foods |  | **98 (77)** | **48 (75)** | **50 (78)** |
|  | Male | 71 (75) | 35 (74) | 36 (75) |
|  | Female | 27 (81) | 13 (76) | 14 (88) |
| Legumes, pulses, and nuts |  | **114 (89)** | **59 (92)** | **55 (86)** |
|  | Male | 84 (88) | 43 (91) | 41 (85) |
|  | Female | 30 (91) | 16 (94) | 14 (88) |
| Eggs |  | **29 (23)** | **14 (22)** | **15 (12)** |
|  | Male | 20 (21) | 10 (21) | 10 (21) |
|  | Female | 9 (27) | 4 (24) | 5 (31) |
| Fruits |  | **7 (5)** | **2 (3)** | **5 (8)** |
|  | Male | 5 (5) | 1 (2) | 4 (8) |
|  | Female | 2 (6) | 1 (6) | 1 (6) |
| Dairy products |  | **23 (18)** | **9 (14)** | **14 (22)** |
|  | Male | 11 (12) | 7 (15) | 4 (8) |
|  | Female | 12 (36) | 2 (12) | 10 (63) |
| Other vegetables |  | **10 (8)** | **2 (3)** | **8 (13)** |
|  | Male | 7 (7) | 1 (2) | 6 (13) |
|  | Female | 3 (9) | 1 (6) | 2 (13) |
| Oil seeds |  | **84 (66)** | **43 (67)** | **41 (64)** |
|  | Male | 61 (64) | 32 (68) | 29 (60) |
|  | Female | 23 (70) | 11 (65) | 12 (75) |
