# Supplementary material for: Dietary variability and micronutrient status of individuals with Yaws infection in Ghana: A case-control study
Source: PLoS One. 2025 Oct 17;20(10):e0334628. doi: 10.1371/journal.pone.0334628 (PMC12533875; doi:10.1371/journal.pone.0334628)
Supplement: S2 Table — (DOCX) [file pone.0334628.s002.docx]

**S2 Table Adequacy of nutrient intake by sex among participants**

| **Variables** | **Frequency** | **Sex** | **All** | **Cases** | **Controls** | **p- value** |
| --- | --- | --- | --- | --- | --- | --- |
|  |  |  | **n = 128 (%)** | **n=64 (%)** | **n=64 (%)** |  |
| Energy (Kcal) | Adequate |  | **22 (17)** | **12 (19)** | **10 (16)** | 0.82 |
|  |  | Male | 17 (18) | 10 (21) | 7 (15) |  |
|  |  | Female | 5 (15) | 2 (12) | 3 (19) |  |
|  | Inadequate |  | 106 (83) | 52 (81) | 54 (84) |  |
|  |  | Male | 78 (82) | 37 (79) | 41 (85) |  |
|  |  | Female | 28 (85) | 15 (88) | 13 (81) |  |
|  |  |  |  |  |  |  |
| Carbohydrate (g) | Adequate |  | **124 (97)** | **62 (97)** | **62 (97)** | >0.99 |
|  |  | Male | 93 (98) | 46 (98) | 47 (98) |  |
|  |  | Female | 31 (94) | 16 (94) | 15 (94) |  |
|  | Inadequate |  | **4 (3)** | **2 (3)** | **2 (3)** |  |
|  |  | Male | 2 (2) | 1 (2) | 1 (2) |  |
|  |  | Female | 2 (6) | 1 (5) | 1 (6) |  |
|  |  |  |  |  |  |  |
| Protein (g) | Adequate |  | **87 (68)** | **43(67)** | **44 (69)** | >0.99 |
|  |  | Male | 65 (68) | 33 (70) | 32 (67) |  |
|  |  | Female | 22 (67) | 10 (59) | 12 (75) |  |
|  | Inadequate |  | **41 (32)** | **21(33)** | **20 (31)** |  |
|  |  | Male | 30 (32) | 14 (30) | 16 (33) |  |
|  |  | Female | 11 (33) | 7 (41) | 4 (25) |  |
|  |  |  |  |  |  |  |
| Fibre (g) | Adequate |  | **10 (8)** | **2(3)** | **8(12)** | 0.10 |
|  |  | Male | 7 (7) | 1 (2.1) | 6 (13) |  |
|  |  | Female | 3 (9) | 1 (5.9) | 2 (13) |  |
|  | Inadequate |  | **118 (92)** | **62(97)** | **56(88)** |  |
|  |  | Male | 88 (93) | 46 (98) | 42 (88) |  |
|  |  | Female | 30 (91) | 16 (94) | 14 (88) |  |
|  |  |  |  |  |  |  |
| Iron (mg) | Adequate |  | **59 (46)** | **29(45)** | **30(47)** | >0.99 |
|  |  | Male | 49 (52) | 26 (55) | 23 (48) |  |
|  |  | Female | 10 (30) | 3 (18) | 7 (44) |  |
|  | Inadequate |  | **69 (54)** | **35(55)** | **34(53)** |  |
|  |  | Male | 46 (48) | 21 (45) | 25 (52) |  |
|  |  | Female | 23 (70) | 14 (82) | 9 (56) |  |
|  |  |  |  |  |  |  |
| Zinc (mg) | Adequate |  | **35 (27)** | **18 (28)** | **17(27)** | >0.99 |
|  |  | Male | 30 (32) | 17 (36) | 13 (27) |  |
|  |  | Female | 5 15) | 1 (5.9) | 4 (25) |  |
|  | Inadequate |  | **93 (73)** | **46(72)** | **47(73)** |  |
|  |  | Male | 65 (68) | 30 (64) | 35 (73) |  |
|  |  | Female | 28 (85) | 16 (94) | 12 (75) |  |
|  |  |  |  |  |  |  |
| Selenium (µg) | Adequate |  | **102 (80)** | **51 (80)** | **51 (80)** | >0.99 |
|  |  | Male | 74 (78) | 37 (79) | 37 (77) |  |
|  |  | Female | 28 (85) | 14 (82) | 14 (88) |  |
|  | Inadequate |  | **26 (20)** | **13 (20)** | **13 (20)** |  |
|  |  | Male | 21 (22) | 10 (21) | 11 (23) |  |
|  |  | Female | 5 (15) | 3 (18) | 2 (13) |  |
|  |  |  |  |  |  |  |
| Vitamin B12 (µg) | Adequate |  | **57 (44)** | **28 (44)** | **29 (45)** | >0.99 |
|  |  | Male | 43 (45) | 22 (47) | 21 (44) |  |
|  |  | Female | 14 (42) | 6 (35) | 8 (50) |  |
|  | Inadequate |  | **71 (56)** | **36 (56)** | **35 (55)** |  |
|  |  | Male | 52 (55)1 | 25 (53) | 27 (56) |  |
|  |  | Female | 19 (58) | 11 (65) | 8 (50) |  |
|  |  |  |  |  |  |  |
| Vitamin C (mg) | Adequate |  | **122 (95)** | **62 (97)** | **60 (94)** | 0.68 |
|  |  | Male | 92 (97) | 47 (100) | 45 (94) |  |
|  |  | Female | 30 (91) | 15 (88) | 15 (94) |  |
|  | Inadequate |  | **6 (5)** | **2 (3)** | **4 (6)** |  |
|  |  | Male | 3 (3) | 0 (0) | 3 (6.3) |  |
|  |  | Female | 3 (9) | 2 (12) | 1 (6.3) |  |
|  |  |  |  |  |  |  |
| Vitamin B6 (µg) | Adequate |  | **112 (88)** | **58 (91)** | **54 (84)** | >0.99 |
|  |  | Male | 85 (89) | 44 (94) | 41 (85) |  |
|  |  | Female | 27 (82) | 14 (82) | 13 (81) |  |
|  | Inadequate |  | **16 (5)** | **6 (9)** | **10 (16)** |  |
|  |  | Male | 10 (11) | 3 (6) | 7 (15) |  |
|  |  | Female | 6 (18) | 3 (18) | 3 (19) |  |
|  |  |  |  |  |  |  |
| Magnesium (mg) | Adequate |  | **79 (62)** | **40 (63)** | **39 (61)** | >0.99 |
|  |  | Male | 59 (62) | 32 (68) | 27 (56) |  |
|  |  | Female | 20 (61) | 8 (47) | 12 (75) |  |
|  | Inadequate |  | **49 (38)** | **24 (37)** | **25 (39)** |  |
|  |  | Male | 36 (38) | 15 (32) | 21 (44) |  |
|  |  | Female | 13 (39) | 9 (53) | 4 (25) |  |
|  |  |  |  |  |  |  |
| Calcium(mg) | Adequate |  | **0** | **0** | **0** | >0.99 |
|  |  | Male | 0 | 0 | 0 |  |
|  |  | Female | 0 | 0 | 0 |  |
|  | Inadequate |  | **128 (100)** | **64(100)** | **64 (100)** |  |
|  |  | Male | 95 (100) | 47 (100) | 48 (100) |  |
|  |  | Female | 33 (100) | 17 (100) | 16 (100) |  |
|  |  |  |  |  |  |  |
| Folic acid (µg) | Adequate |  | **0** | **0** | **0** | >0.99 |
|  |  | Male | 0 | 0 | 0 |  |
|  |  | Female | 0 | 0 | 0 |  |
|  | Inadequate |  | **128 (100)** | **64(100)** | **64 (100)** |  |
|  |  | Male | 95 (100) | 47 (100) | 48 (100) |  |
|  |  | Female | 33 (100) | 17 (100) | 16 (100) |  |
