# Supplementary material for: Dietary variability and micronutrient status of individuals with Yaws infection in Ghana: A case-control study
Source: PLoS One. 2025 Oct 17;20(10):e0334628. doi: 10.1371/journal.pone.0334628 (PMC12533875; doi:10.1371/journal.pone.0334628)
Supplement: S4 Table — (DOCX) [file pone.0334628.s004.docx]

**S4 Table Dunn’s multiple comparison for statistically significant micronutrient (Selenium) within lesion type**

| Dunn's multiple comparisons test | Mean rank difference | Significant? | Adjusted p-value |
| --- | --- | --- | --- |
| Ulcer vs. squamous macules | -9.970 | No | 0.58 |
| Ulcer vs. plantar | -25.68 | Yes | 0.01 |
| Ulcer vs. papilloma | -3.512 | No | >0.99 |
| Squamous macules vs. plantar | -15.71 | No | 0.52 |
| Squamous macules vs. papilloma | 6.458 | No | >0.99 |
| Plantar vs. papilloma | 22.17 | No | 0.52 |
